# Immunomodulatory activity of manganese dioxide nanoparticles: Promising for novel vaccines and immunotherapeutics

**DOI:** 10.3389/fimmu.2023.1128840

**Published:** 2023-02-28

**Authors:** Yuhe Huang, Yongdui Ruan, Yuhe Ma, Dongsheng Chen, Tangxin Zhang, Shuhao Fan, Wensen Lin, Yifan Huang, Hongmei Lu, Jun-Fa Xu, Jiang Pi, Biying Zheng

**Affiliations:** ^1^ Guangdong Provincial Key Laboratory of Medical Molecular Diagnostics, The First Dongguan Affiliated Hospital, Guangdong Medical University, Dongguan, China; ^2^ Institute of Laboratory Medicine, School of Medical Technology, Guangdong Medical University, Dongguan, China; ^3^ Dongguan Key Laboratory of Environmental Medicine, School of Public Health, Guangdong Medical University, Dongguan, China

**Keywords:** manganese dioxide nanoparticles, manganese ion, immune regulation, vaccines, immunotherapies

## Abstract

Manganese (Mn), a nutrient inorganic trace element, is necessary for a variety of physiological processes of animal body due to their important roles in oxidative regulation effects and other aspects of activities. Moreover, manganese ion (Mn^2+^) has widely reported to be crucial for the regulations of different immunological responses, thus showing promising application as potential adjuvants and immunotherapeutics. Taking the advantages of Mn-based biological and immunological activities, Manganese dioxide nanoparticles (MnO_2_ NPs) are a new type of inorganic nanomaterials with numerous advantages, including simple preparation, low cost, environmental friendliness, low toxicity, biodegradable metabolism and high bioavailability. MnO_2_ NPs, as a kind of drug carrier, have also shown the ability to catalyze hydrogen peroxide (H_2_O_2_) to produce oxygen (O_2_) under acidic conditions, which can enhance the efficacy of radiotherapy, chemotherapy and other therapeutics for tumor treatment by remodeling the tumor microenvironment. More importantly, MnO_2_ NPs also play important roles in immune regulations both in innate and adaptive immunity. In this review, we summarize the biological activities of Manganese, followed by the introduction for the biological and medical functions and mechanisms of MnO_2_ NPs. What’s more, we emphatically discussed the immunological regulation effects and mechanisms of MnO_2_ NPs, as well as their potentials to serve as adjuvants and immunomodulators, which might benefit the development of novel vaccines and immunotherapies for more effective disease control.

## Introduction

As an essential trace element for human body, manganese (Mn) plays an important role in promoting the normal growth and development of bones, maintaining normal glucose, lipid metabolism and the function of central nervous system. Manganese is also an important component and activator of some critical enzymes in the body that regulate oxidative stress (1), antioxidant status (2), mitochondrial function (3) and neurotransmitter synthesis (4). Moreover, manganese ions (Mn^2+^) has also shown promises as a functional intracellular MR imaging contrast agent through its ability to evaluate cellular integrity, activity, and neural connectivity (5, 6). Taking the advantages of these important roles in human health, manganese-based compounds and materials have been considered as a kind of potential candidates for novel diagnosis strategy, vaccine and drug development.

Interestingly, increasing evidences are indicating that manganese can regulate the complicated immunological responses in different conditions (7, 8). The immune function of manganese has been introduced in terms of nutritional immunity (9) due to its roles as a nutrient inorganic micronutrient necessary for a variety of physiological processes, including metabolism, antioxidant defense and antibody production (10, 11). Although the precise mechanisms are still needed to be further investigated, manganese homeostasis has been proved to be critical for different immune responses, especially for its role as an alarm protein of innate immunity for host anti-cancer and anti-infection defense (12). And based on the ability to control innate immunity, manganese has also been proved to possess strong function for the regulation of adaptive immunity.

Nanotechnology is defined as the science and engineering involved in the design, synthesis, characterization and application of materials and devices with molecular precision (13), which is based on a variety of traditional science, including chemistry, physics, materials science, biology and medicine (13, 14). Nanotechnology has demonstrated plenty of applications in biology and medicine, including *in situ* and multimodal imaging, gene/protein/drug delivery and immunological regulations, which therefore shows promising potentials for diagnostic, vaccine and therapeutic strategy development (15, 16).

Manganese can also be designed into functional nanomaterials with different manganese valence with different associated functions. And among these manganese- based nanomaterials, manganese dioxide nanoparticles (MnO_2_ NPs) are one of the most stable and functional nanomaterials with some important biological activities and immunological response regulatory activities (5). Under acidic conditions, MnO_2_ NPs can efficiently catalyze the production of O_2_ by interact with H_2_O_2_ to release Mn^2+^ due to its peroxidase-like activity (17). Additionally, MnO_2_ NPs can also interact with the intracellular glutathione (GSH) to release Mn^2+^ (18, 19). These abilities not only allow MnO_2_ NPs to regulate the cellular oxidative stress, but also endow MnO_2_ NPs the potentials to regulate multiple immunological responses by the oxidative associated mechanisms and Mn^2+^ associated mechanisms (7, 20).

In this review, we summarized the basic biological functions of manganese and MnO_2_ NPs, as well as their relevant biological and medical application. And due to the increasing attentions paid onto the immune therapy and vaccine development, we emphatically discussed the immunological regulation effects of MnO_2_ NPs, followed by their promising application in different diseased conditions, which is expected to benefit the future development of novel vaccines and therapies based on MnO_2_ NPs.

## Biological activity of MnO_2_ NPs

### MnO_2_ NPs can be used as carrier for drug/nucleic acid/protein delivery

As a kind of typical nanomaterial MnO_2_ NPs can be used as carriers to design targeted and controlled delivery systems. MnO_2_ NPs with mesoporous shell have been widely proved to show the ability to load chemotherapy drugs for efficient targeted drug delivery with controlled drug release behaviors (21, 22). MnO_2_ NPs or MnO_2_ nanosheets can also be used as the carrier of the photosensitizer drugs to achieve the controlled intracellular release for enhanced phototherapy efficiency (23). The main role of MnO_2_ nanocarriers for drug delivery is as a “gatekeeper” for the encapsulated drugs, which decompositions at the acidic pH after reaching the targeted site to release the encapsulated drugs, thereby improving the bioavailability, efficacy selectivity of drugs for more effective therapy (19).

Except for the drug carrier roles, MnO_2_ NPs can also be used as nucleic acid vector for nucleic acid delivery-based therapies. For example, MnO_2_ nanomaterials can be used also as siRNA loading system for targeted delivery and controlled release siRNA for more effective gene expression regulation, which therefore allowed tumor-related gene therapy (24, 25). In addition to the ability to load siRNA, MnO_2_ nanomaterials can also be used as DNA nanocarrier, which allows the conjugation of DNA aptamers onto the system to achieve the binding of DNA aptamers to their targets for more effective therapy and diagnostic strategy developments (26, 27). Additionally, DNA enzyme can also be adsorbed onto MnO_2_ nanomaterials to effectively deliver DNAzyme into cells (23, 28), which enables DNAzyme to catalyze multiple reactions (e.g., RNA or DNA cutting and linking or DNA phosphorylation) for gene therapy (29). Moreover, MnO_2_ NPs can also be used to wrap proteins, which can combine the advantages of engineered proteins and nanocarriers to provide an intelligent strategy to efficiently deliver functional proteins *in vivo *(30). The advanced property of MnO_2_ NPs to serve as drug/nucleic acid/protein carriers provide novel possibilities for the development of more effective therapeutics and diagnostics.

### MnO_2_ NPs enhanced T1-weighted MRI

MnO_2_ NPs can also be used as a T1-weighted magnetic resonance imaging (MRI) probe to significantly enhance MRI signals. The Mn^2+^, which can be generated by MnO_2_ NPs decomposition, has been approved by FDA as a clinical T1-weighted magnetic resonance (MR) imaging contrast agent. Mn^2+^ improves the signal intensity and specificity of MRI by shortening T1 relaxation, resulting in efficient positive contrast enhancement and brighter images. MnO_2_ NPs have been widely proved to release Mn^2+^ after their entry into cells by reacting with H_2_O_2_ or GSM. Thus, MnO_2_ NPs are also expected to be used as T1-weighted MRI probes due to their ability to release Mn^2+^, which provides new possibility to develop MnO_2_ NPs into novel diagnostic and therapeutic agents (5, 31).

### MnO_2_ NPs remodel tumor microenvironment

**Figure 1:**
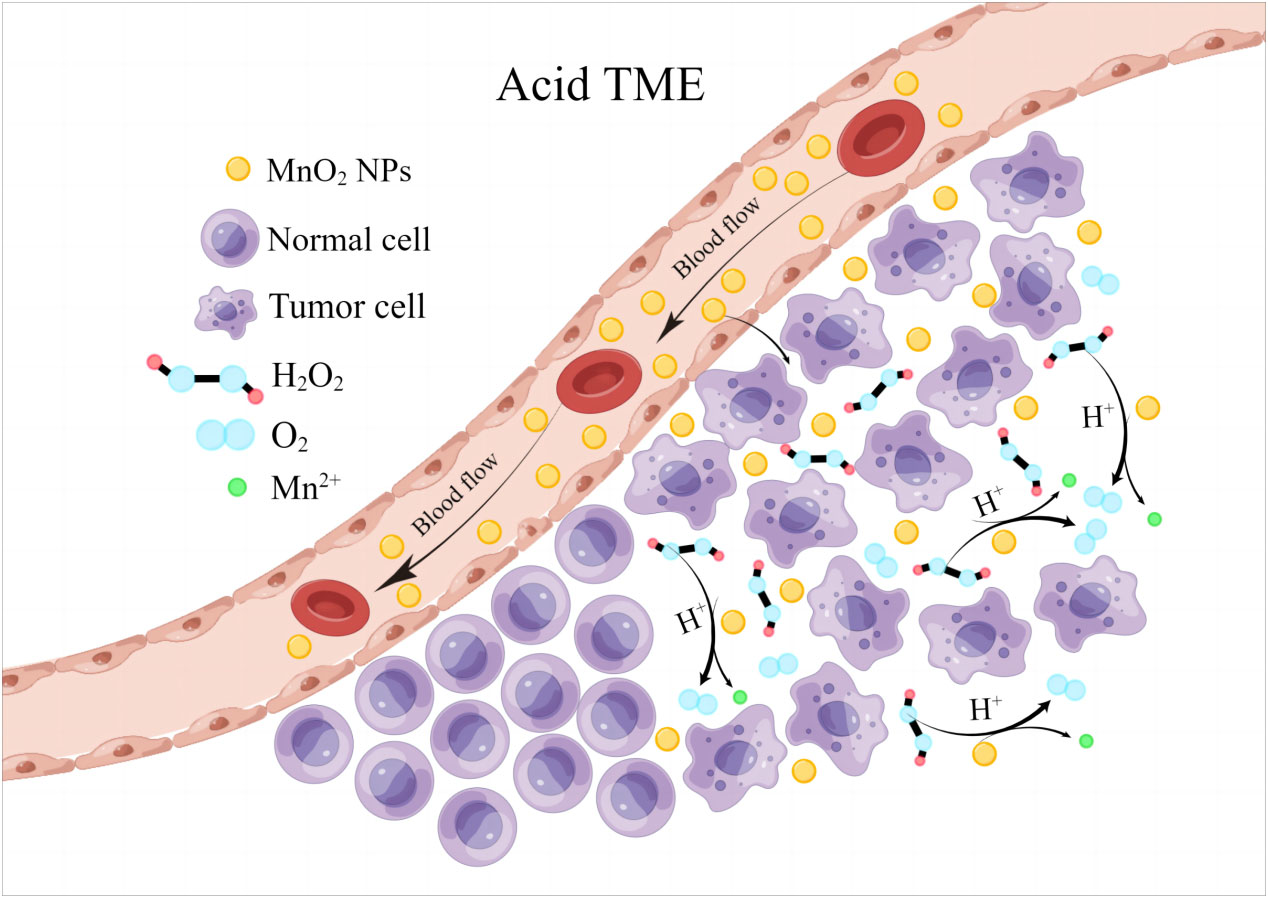
MnO_2_ NPs remodel tumor microenvironment by reacting with H_2_O_2_ to release oxygen and Mn^2+^.

Taking the advantages of the outstanding biological activities of Mn, MnO_2_ NPs have also been proved to show plenty of biological functions that directly regulate tumor growth. Tumor microenvironment (TME) is typically characterized by its hypoxia and acidic pH, where cancer cells can produce large amounts of H_2_O_2_ and GSH for their metabolism and resistance to immunological killings. Under the acidic conditions of TME, MnO_2_ NPs can effectively catalyze the *in situ* production of O_2_ by reacting with endogenous H_2_O_2_ to release Mn^2+^ due to its peroxidase-like activity, thus significantly relieving tumor hypoxia (**Figure 1**).

**Figure 2:**
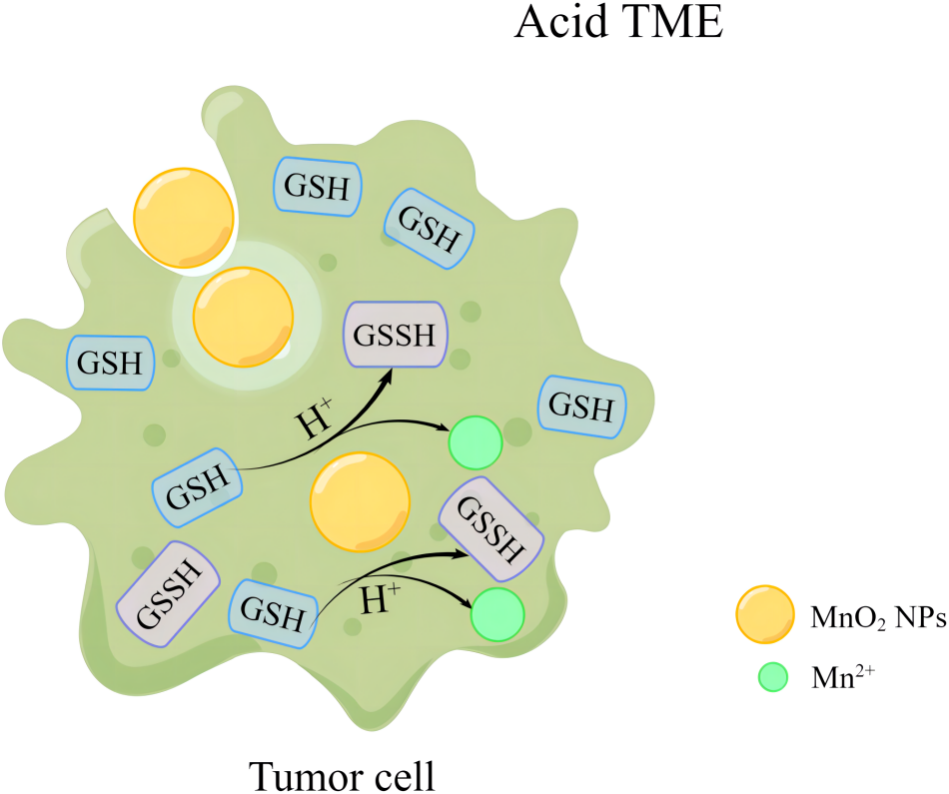
MnO_2_ NPs remodel tumor microenvironment by reacting with GSH to release Mn^2+^.

Similarly, MnO_2_ NPs can react with GSH in tumor cells to generate GSSH and Mn^2+^, which would result in the in uncontrolled reactive oxygen species (ROS) levels in tumor cells due to the decreased GSH level (**Figure 2**). The ability of MnO_2_ NPs to regulate TME by catalyzing REDOX activity therefore allows MnO_2_ NPs to enhance the sensitivity of anti-tumor therapy (17, 32, 33).

In addition, viral or bacterial infections are often accompanied by inflammation.ROS production increases in tissues under inflammation, and the concentration of ROS such as H_2_O_2_ can be up to more than 100 times that of normal tissues (34, 35). Besides, hypoxia is a common microenvironmental feature in the inflammatory process related to bacterial infection (36), especially in the site of abscess caused by infection and the bacterial biofilm of chronic wounds, which is characterized by hypoxia, acidic pH and high H_2_O_2_ content (37, 38). MnO_2_ NPs can catalyze H_2_O_2_ to produce O_2_ and release Mn^2+^ under acidic conditions and even physiological conditions (pH=7.4) (39, 40), so MnO_2_ NPs can continue to produce oxygen and improve oxidative stress, thus reducing inflammation associated with infection.Therefore, it is believed that MnO_2_ NPs can also reshape the environment of the infected site to a certain extent.

### MnO_2_ NPs enhance the efficacy of different therapies

Phototherapy, including photodynamic therapy (PDT) and photothermal therapy (PTT), relies on photosensitizers (PS) or photothermal agents (PTA) to convert external light energy into monomorphic oxygen (O_2_) and heat for direct tumor cell killings. Phototherapy is heavily dependent on the production of O_2_, and the hypoxia of TME will greatly limit the efficacy of phototherapy. The REDOX function of MnO_2_ NPs can overcome the hypoxia of TME, thus improving the efficacy of phototherapy against tumor (17, 41). Moreover, phototherapy can also activate acute inflammation or induce immunogenic cell death (ICD) to enhance tumor immunogenicity, which could turn the non-immunogenic tumor into the more sensitive immunogenic tumor for immunotherapy (42, 43). Sonodynamic therapy (SDT), similar to PDT, can activate sound sensitizers to produce ROS and overcome the limitation of light penetration depth in phototherapy. However, SDT is also limited by the hypoxic TME, which can also mediate the resistance of tumor to radiotherapy (RT). Based on the ability to catalyze REDOX activity, MnO_2_ NPs can also be applied to enhance the efficacy of SDT and RT for tumor treatment (44–46).

### MnO_2_ NPs as a CDT agent can improve the chemodynamic therapy performance

Interestingly, by regulating REDOX, MnO_2_ NPs can decompose and release Mn^2+^, which makes MnO_2_ NPs can be used as a CDT agent. L/D-MnO_2_@Pt NPs could be specifically internalized by tumor cells and efficiently deplete the glutathione (GSH) through redox reaction to release Mn^2+^, while the released Mn^2+^ could exhibit strong chemodynamic effects through Fenton-like reactions for enhanced tumor therapy efficiency (32). Similarly, stimuli-responsive manganese carbonate-indocyanine green complexes (MnCO_3_-ICG) can also release “ion reactors” of Mn^2+^ and ICG in acidic tumor environment, which accelerated the generation of hydroxyl radicals for the oxidative stress damage of tumors cells by catalyzing Mn-mediated Fenton-like reaction to suppress tumor growth more effectively (47). These results strongly suggested that MnO_2_ NPs based drug delivery system not only introduce the enhanced targeting effects of drugs, but also allowed MnO_2_ NPs to be used as a CDT agent to improve the catalytic efficiency of Fenton-like reaction to improve CDT performance (48).

### MnO_2_ NPs have great biocompatibility

As mentioned above, MnO_2_ NPs have many biological properties, which might be very beneficial for the treatment of diseases. The toxicity and biocompatibility of MnO_2_ NPs are the most critical issues if MnO_2_ NPs are to be effectively and practically used in clinical treatment. Interestingly, the whole-body biocompatibility of MnO_2_ NPs has been demonstrated in tumor models (49). Moreover, some literatures have shown that MnO_2_ has a crucial advantage -it can be decomposed to release water-soluble Mn^2+^ in the presence of H_2_O_2_ under acidic conditions, and more importantly, Mn^2+^ can be easily and quickly filtered out of the body by the kidney under physiological conditions (39, 50). Some related works also showed that even when high doses of MnO_2_ NPs were injected into healthy mice, their liver function markers, kidney function markers and blood routine indexes were no different from normal values, suggesting that MnO_2_ NPs did not cause significant liver and kidney dysfunction *in vivo* (40). Thus, unlike many other non-biodegradable inorganic nanomaterials, MnO_2_ NPs are a kind of biodegradable nanomaterials that can be used *in vivo* without long-term toxicity concerns. In addition, MnO_2_ NPs can also be modified with some good biocompatible and biodegradable materials, such as glycol chitosan (GC) polymer and polyethylene glycol (PEG) (18). Loading MnO_2_ NPs with GC or PEG can further enhance the biocompatibility of nanoparticles and improve their colloidal stability (30, 49). In addition, albumin, such as human albumin (HSA) and bovine albumin (BSA), also have excellent biocompatibility and low immunogenicity, which make them very suitable for wrapping MnO_2_ NPs (41, 51). Moreover, modification of nanoparticles with hyaluronic acid (HA) or folic acid (FA) can also make MnO_2_ NPs more biocompatible in treatment (30, 31).

In addition, MnO_2_ NPs also have great potential for immunotherapy or vaccine development due to the key role of Mn^2+^ in triggering innate immunity and enhancing adaptive immunity. In conclusion, MnO_2_ NPs can be used as a kind of novel agents to deliver drugs, regulate REDOX, regulate TME, increase MRI signals, enhance the efficiency of photodynamic therapy, photothermal therapy, sonodynamic therapy, radiotherapy and as a CDT agent to improve chemodynamic therapy performance, activate innate and adaptive immunity with high biocompatibility, which therefore allow MnO_2_ NPs to be used for novel vaccine, diagnostic, therapeutic, therapeutic vaccine or diagnosis-therapy combined strategy development.

### Innate immune regulation of MnO_2_ NPs

MnO_2_ NPs have the ability to regulate ROS levels due to the peroxidase activity of MnO_2_ (17), and the released Mn^2+^ from MnO_2_ NPs by catalyzing REDOX reactions also has the ability to regulate ROS levels (32). Additionally, MnO_2_ can also act as a kind of novel oxidants to consume excess GSH production in tumors to reduce ·OH depletion and release Mn^2+^ (18, 32). The released Mn^2+^ can in turn mediate Fenton-like reactions by catalyzing the generation of highly active ·OH from H_2_O_2_ (32) to induce high oxidative stress in tumor cells and the production of proinflammatory factors, such as TNF-α (52–55). The high oxidative stress burdens could induce the damage of tumors cells, which is helpful for the tumor antigens release to stimulate anti-tumor immune responses (56–58). And the increased production of proinflammatory factors could induce acute inflammation in tumor, which can stimulate the maturation of DC (7), and thus further enhance the body’s innate anti-tumor immune responses. These effects allow MnO_2_ NPs to remodel TME and enhance the immunogenicity of tumor cells for enhanced anti-tumor immunity, which suggested MnO_2_ NPs as immunomodulators and immune activators to regulate innate anti-tumor immune response. Moreover, the inflammatory microenvironment caused by infection is also characterized by hypoxia, acidic pH, accumulation of lactic acid and overexpression of ROS. The functions of MnO_2_ NPs are also helpful to reverse these microenvironment associated inflammatory responses (59–62). Therefore, MnO_2_ NPs are also expected to play important roles in innate immune defense against infections.

### MnO_2_ NPs promote the infiltration and activity of innate immune cells

It’s well known that tumor cells produce a large amount of lactic acid due to high metabolism and insufficient blood supply in solid tumors, and they also produce large amounts of GSH as antioxidants to cause hypoxia and acidic pH, which are recognized as the characteristics of TME (18, 41, 63). Hypoxia not only causes the accumulation of immunosuppressive metabolites (such as adenosine and lactic acid) in solid tumors, but also restricts the infiltration of immune cells, thus inhibiting the body’s anti-tumor immune responses. For example, the accumulated lactic acid induced by hypoxia could reduce antigen presentation and inhibit NK cell activity by inhibiting DC differentiation, which would further promote the infiltration of M2-TAMs (tumor-associated macrophages) and other immunosuppressive cells into the solid tumors (7).

Hypoxia helps tumor cells recruit macrophages, and the recruitment of tumor-associated macrophages (TAMs) into avascular areas sustains tumor progression (64). Classic M1 phenotype macrophages is a type of proinflammatory cell with anti-proliferative and cytotoxic activity due to its ability to secrete reactive nitrogen and reactive oxygen species (e.g., hydrogen peroxide and NO) (31). Moreover, M1 can directly engulf tumor cells (65) or target tumor cells by releasing pro-inflammatory cytokines (66), and can also act as APC to activate T cells, thus enhancing adaptive anti-tumor immunity (67, 68). However, the continued action of TME can polarize M1 into M2-phenotypic macrophages. In contrast to M1 macrophages, M2 macrophages promote immunosuppression by producing various cytokines and growth factors (69), and can promote tumor cell survival in fragile environments such as TME or infection by limiting alternating activation of interleukin-13 and 4 (70, 71). Interestingly, the alleviating effect of MnO_2_ NPs on TME hypoxia can repolarize the M2 phenotype of TAM to the classic M1 phenotype (72, 73), while the released Mn^2+^ from MnO_2_ NPs also promotes M2 macrophages to M1 macrophages by activating interferon signaling (67).

**Figure 3:**
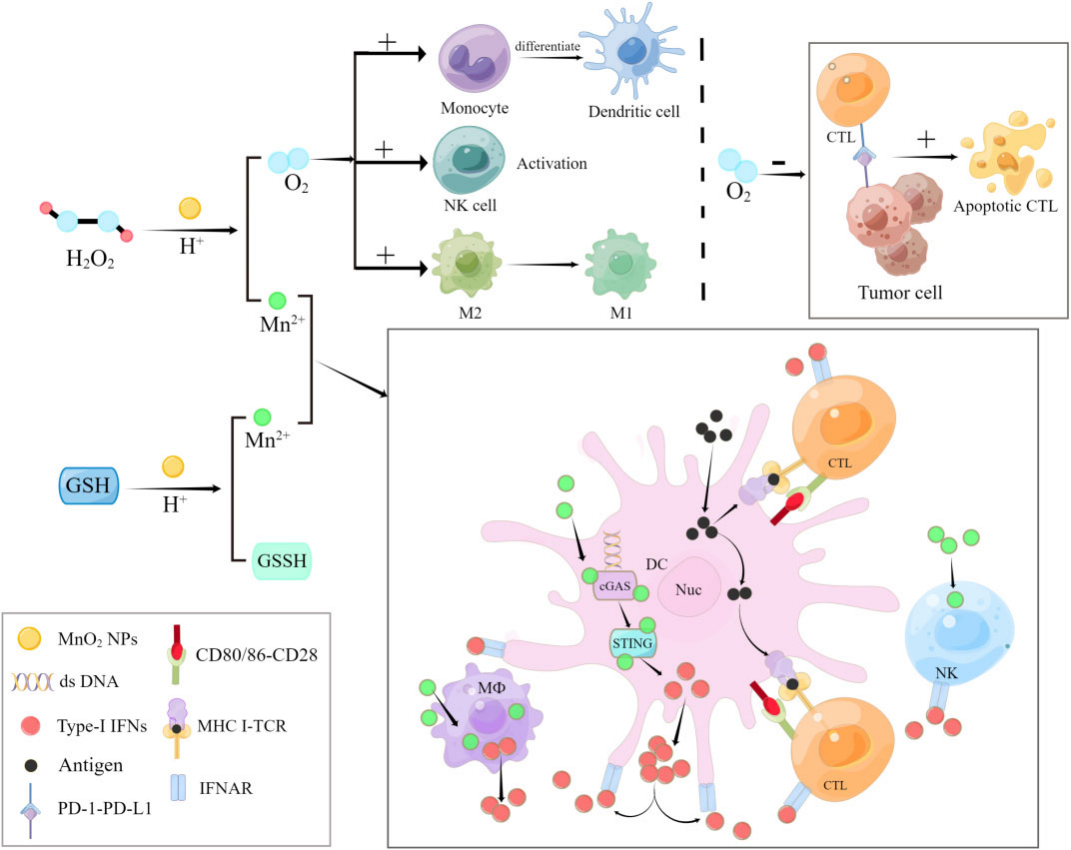
MnO_2_ NPs regulate innate and adaptive immune cells against tumor and infections.

DC is the main supervisor of the immune system (74). DC can recognize, capture and treat the antigen of pathogens at the invasion site, convert the antigen into polypeptides and present the antigenic peptide (75) to cytotoxic CD8+T(CTL) cells and pro-inflammatory CD4+T cells (Th1). Meanwhile, DC also secretes IL-12 (76), which plays an important role in the growth and activity of NK cells and T cells. However, the acidic pH of TME and the accumulation of immunosuppressors (e.g., lactic acid) inhibited the immunostimulatory function of DC (77, 78) and limited the differentiation of monocytes into DC (63). MnO_2_ NPs can also block the production of lactic acid while increasing the production of oxygen (79), which not only enhances the mature activation of DC (80) but also reverses the inhibitory effect of lactic acid on DC (65). By targeting hypoxia of TME immunosuppression, MnO_2_ NPs can exert their peroxide-like activity to react with tumor endogenous H_2_O_2_, which could generate *in situ* O_2_ to relieve TME hypoxia to remit the immune suppression in solid tumors (33, 81). The remission of hypoxia can remodel TME (82), which can also promote the differentiation of monocytes into DC (67), promote the transformation of M2 into anti-tumor immune type M1 (22, 67, 73), and enhance the anti-tumor activity of NK cells (65, 83).By enhancing the anti-tumor activity of immune cells and promoting the infiltration of immune cells in solid tumors, MnO_2_ NPs can significantly enhance the innate immunological responses against tumor (**Figure 3**).

Tissue hypoxia and lactic acid buildup due to hypoxia are common microenvironmental features of inflammatory processes associated with bacterial infections (84–86). Meanwhile, H_2_O_2_ is also overexpressed in the inflammatory microenvironment. The peroxidase-like activity of MnO_2_ NPs also catalyzes H_2_O_2_ to relieve tissue hypoxia in inflammatory microenvironments and inhibits lactic acid production by producing *in situ* oxygen (60–62). MnO_2_ NPs can enhance the effects of DC and macrophages by remodeling inflammatory microenvironment, and enhance the innate immunity against bacteria infections (**Figure 3**). More importantly, some H_2_O_2_-producing bacteria, such as Streptococcus pneumoniae and Streptococcus oral, release large amounts of H_2_O_2_ to oxidatively inactivate inflammatosomes in immune cells (87). Moreover, excess ROS production by immune cells is also associated with the decreased activation of inflammasome (88–91). However, inflammasome is a substance that plays a crucial role in the body’s innate immunity against bacterial pathogens (88, 92, 93). Therefore, it is reasonable to speculate that MnO_2_ NPs might also inhibit the decreased activation of inflammasome by clearing excessive H_2_O_2_, and indirectly promote the body’s anti-bacterial innate immune responses. Other studies have also shown (94) that MnO_2_ nanosheets can directly contact with the bacterial cell membrane and produce a large amount of ROS(mainly ·O_2_-) at the contact site, which would cause damage to the cell membrane, resulting in leakage of electrolytes/intracellular contents and reduction ATPase activity for bacterial death.

The relief of MnO_2_ NPs in hypoxic environment enhanced the activity of macrophages, DC and NK cells. Macrophages and DC can secrete IFN-I, and Mn^2+^ released by MnO_2_ could promote the production of IFN-I through the cGAS-STING pathway in the presence of viral infection (95). IFN, a group of signaling proteins produced and secreted by host cells in response to pathogen infection (96, 97), has been used in the treatment of hepatitis B virus infection (98–100). IFN-I could induce multiple innate immune responses, such as limiting virus invasion, replication, assembly, and transmission (67). More importantly, the hypoxic state facilitates the transition of Epstein-Barr virus (EBV,HHV-4) and Kaposi Sarcoma-associated herpes virus (KSHV,HHV-8) from latent to dissolved mode (101). And hypoxia could increase the expression of Zta, a protein that mediates the transition between latent and lytic EBV infection, which could in turn increase the amount of viral DNA replication in infected cells (102). Therefore, the ability of MnO_2_ NPs to increase IFN-I production and relief the hypoxic environment by oxygen production also provides novel possibilities to enhance the innate immune response against viruses.

### MnO_2_ NPs promote innate immunological responses by regulating cGAS-STING signaling pathway

**Figure 4:**
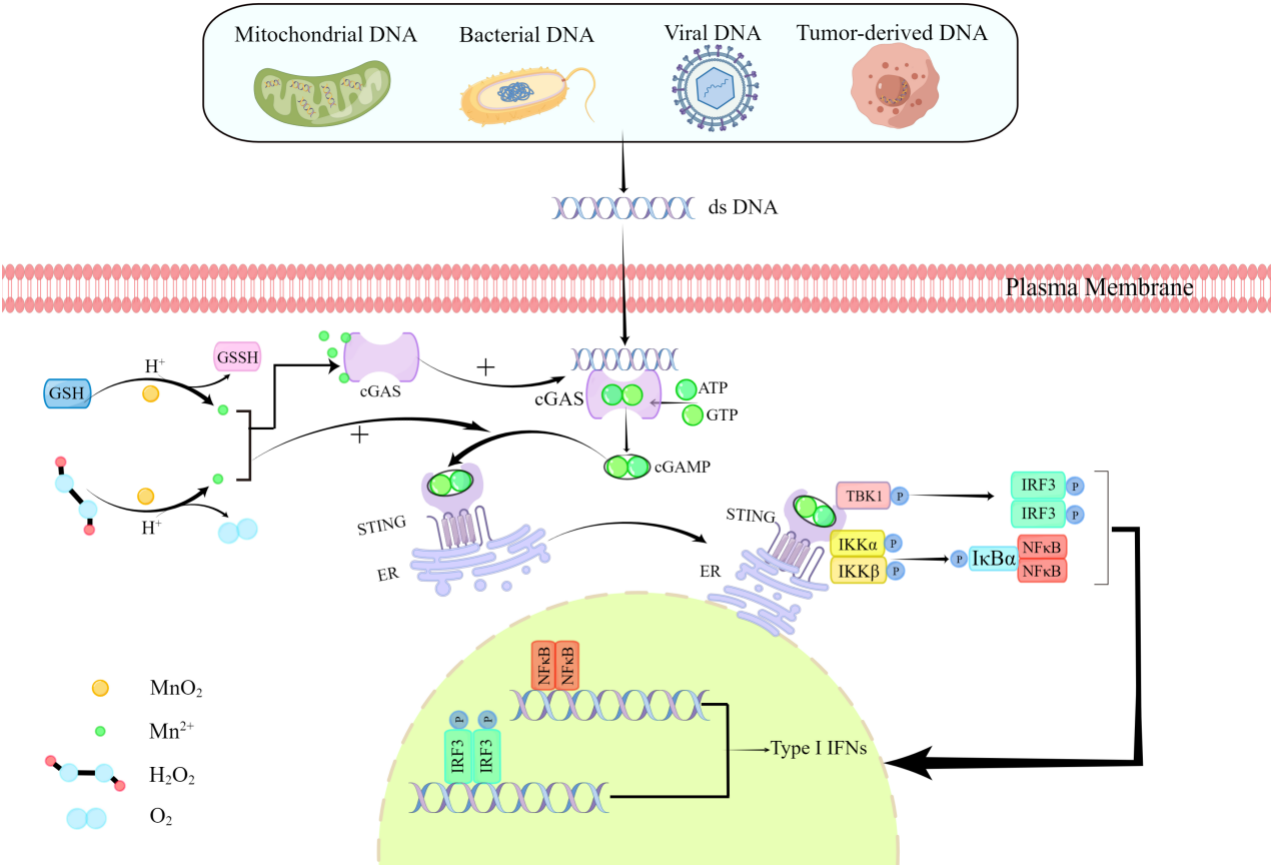
MnO_2_ NPs activate cGAS-STING signaling pathway by releasing Mn^2+^.

The released Mn^2+^ from MnO_2_ NPs has also been proved to show critical roles in immune regulations in different diseased conditions. After DNA virus infection, host cells release Mn^2+^ from membrane organelles to cytosol, which is accumulated to activate cGAS-STING signaling pathway to induce IRF3 phosphorylation, activate NF-κB pathway and promote IFN-I production for antiviral effects. The cGAS-STING pathway can also be activated by tumor-derived dsDNA to induce IFN-I, which can promote the maturation/activation of tumor-infiltrating dendritic cell (DCs) or macrophages and enhance the presentation of tumor-specific antigens to activate CD8+T cells and NK cells for tumor immune responses (41, 42). Therefore, Mn^2+^ released by MnO_2_ NPs after catalytic REDOX can also trigger innate immunity by activating cGAS-STING signaling pathway. cGAS can be activated by any dsDNA in a sequence independent manner to activate cGAS-STING signaling pathway, which has been reported to be a critical pathway for anti-tumor, antiviral and anti-bacterial defense (53, 103). dsDNA can be detected by the membrane localization protein cGAS (cyclic GMP-AMP(cGAMP) synthetase) (104), leading to the synthesis of the second messenger cGAMP and its binding to STING(stimulator of interferon gene), followed by activation of interferon regulator IRF3 and transcription factor NF-kB. These effects would result in the expression of proinflammatory factors and IFN-I (53, 105), which, in addition to its effects, can promote the maturation and activation of DC and macrophages and enhance NK cell activity (106), thereby further enhancing the innate immune responses (**Figure 4**). However, Mn^2+^ itself has been reported to be an effective innate immune stimulator by activating cGAS-STING pathway. Even if there is only a low level of dsDNA (less than 10^4^mg/ml) or even no dsDNA in the cytoplasm, Mn^2+^ can activate the cGAS-STING signaling pathway to play the similar anti-tumor, antiviral and anti-bacterial roles (107). Mn^2+^ may promote cGAS-STING pathway activation through two mechanisms. Firstly, Mn^2+^ can directly bind to cGAS and induce the activation of cGAS protein to form a compact conformation that is more easily to be further activated. The direct interactions between Mn^2+^ and cGAS proteins may enhance the sensitivity and enzymatic activity of cGAS to dsDNA, which makes very low concentration of dsDNA can also activate cGAS and initiate noncanonical 2’3’ -cGAMP synthesis as a second messenger (108).The second potential mechanism is the ability of Mn^2+^ to enhance the affinity of cGAMP and STING on the surface of the ER, which could finally enhance the activity of STING (9) and promote the activation of cGAS at the same time (9, 41).

In addition, activation of cGAS by Mn^2+^ can also alarm the immune system to inhibit cancer development by promoting tumor cell senescence and cytokine production (103). And based on these results, cGAS-STING agonists are therefore being developed as a novel cancer therapeutic, and a greater understanding of cGAS-STING pathway regulation is leading to a broadened list of candidate immune regulatory targets (109). Therefore, MnO_2_ NPs, with the ability to produce Mn^2+^, can act as novel cGAS-STING agonists to ameliorate innate immune responses.

### MnO_2_ NPs induced ICD

Some studies have also identified the intrinsic immune regulatory properties of MnO_2_ NPs as a unique inducer of nutritionally responsive immunogenic cell death (ICD). While ICDs can directly kill tumor cells, they also highly expose damage-related molecular patterns (DAMPs) on tumor cells (110–112). Lots of evidences support the notion that DAMPs contribute to adaptive immunity in vertebrates (113, 114), and the exposure or release of DAMPs is a key event in the initiation of adaptive immunity (115, 116). DAMPs can not only bind to pattern recognition receptors (PRR) to subsequently promote adaptive immunity by inducing DC activation, but can also enhance adaptive immune responses by affecting the presentation of antigens associated with dying cells, promoting phagocytic action (“ eat-me signaling, “e.g., calreticulin), and facilitating antigen extraction and processing (“ present-me signaling,” e.g., F-actin or HMGB1) (111, 115, 117).

MnO_2_ nanoparticles have been widely employed in cancer immunotherapy, playing a subsidiary role in assisting immunostimulatory drugs by improving their pharmacokinetics and/or creating a favorable microenvironment. Moreover, Yang et al. introduced an intrinsic immunomodulatory property of MnO_2_ NPs as a unique nutrient-responsive immunogenic cell death (ICD) inducer, capable of directly modulating immunosurveillance toward tumor cells (110). The underlying mechanism of MnO_2_ NPs-mediated selective ICD induction might be associated with the concurrently upregulated oxidative stress and autophagy. This starvation- immunotherapy method based on MnO_2_ NPs is realized by the cut off intratumoral nutrient supply, eliciting potent efficacy for suppressing local and distant tumors. Their findings open up a new avenue toward biomedical applications of MnO_2_ NPs by manipulating ICD, enabling an innovative therapeutics paradigm with great clinical significance.

### Adaptive immunoregulatory function of MnO_2_ NPs

By activating innate immune responses, MnO_2_ NPs can further promote innate immune response and adaptive immunity for disease treatment. It’s widely known that hypoxia helps tumor cells to evade the body’s adaptive immune responses. Under hypoxia, accumulation of hypoxia-inducable factor-1 (HIF-1) activates hypoxia- adapted genes, which induces the expression of the immunosuppressive molecule PD-L1 in tumor cells, thereby increasing the resistance of tumor cells to cytotoxic lymphocyte (CTL)-mediated lysis and inducing CTL apoptosis (118–123). MnO_2_ NPs can catalyze tumor-derived H_2_O_2_ to produce O_2_ *in situ* under the acidic pH environment in TME to alleviate hypoxia (22, 23, 45, 124, 125), which could inhibit immune evasion promoted by hypoxia, and indirectly promote adaptive immunity against tumor cells (67, 72, 126, 127) (**Figure 3**). By controlling Mn^2+^, MnO_2_ NPs can also activate cGAS-STING signaling pathway to trigger adaptive immune responses through the antigen presentation by APCs. It is well known that APCs are the bridge between innate immunity and adaptive immunity, among which DC plays a critical role (74, 80). Mn^2+^ can induce the production of IFN-I by activating cGAS-STING pathway to promote the maturation and activation of tumor-infiltrating macrophages and DCs, and enhance the presentation of tumor-specific antigens (126), which is a key step in activating CD8+T cells and increasing the proportion of memory CD8+T cells (128, 129). IFN-I can also up-regulate costimulatory molecules on the surface of APCs, such as CD80 and CD86 (76, 105). Costimulatory molecules bind to the receptor CD28 protein on the surface of T cells to stimulate the activation, proliferation and differentiation of tumor-specific T cells into memory T cells. This also has significant implications for the treatment of abscesses caused by bacterial infections, given that the immune mimicking effects of Mn^2+^, which can be released by MnO_2_ NPs, to induce robust immune memory (38). Similarly, the adaptive immunity triggered by APCs is of great significance to the body’s antibacterial activity. When the host needs to resist pathogen invasion, DC could present antigenic peptides to CD8+T cells and Th1 cells to activate T lymphocytes through direct cell-cell contact (75) (**Figure 3**). Studies have shown that Mn^2+^ promotes HBs–AG antibody, ALT, and IFN-β production after HBs–AG immunization to regulate antigen presentation and CD4+T cell activation (95). Moreover, IFN-I induced by Mn^2+^ is also involved in the regulation of cellular and humoral immunity mediated by CD8+T cells (130), further suggesting that MnO_2_ NPs might be beneficial for the adaptive immunity against viruses by regulating cellular and humoral immunity. In conclusion, MnO_2_ NPs can be used as a kind of novel immunological modulators to enhance innate immune responses, increase the efficacy of ICD inducers and improve adaptive immune responses by overcoming hypoxia, regulating ROS levels and controlling cGAS-STING signaling pathway. The ability of MnO_2_ NPs to release Mn^2+^ has been regarded as one of the key reasons to activate innate immune responses and trigger adaptive immunity through APCs, which is therefore expected to show promising applications in future vaccine and therapeutic strategy development.

## Conclusion and perspectives

In the past few decades, the research on the biological and medical application of MnO_2_ NPs has been rapidly developed to show many profound advances. Here, we have reviewed the biological activities of Mn^2+^ and MnO_2_ NPs, especially their immunological regulation effects, as well as their potential application. Due to the important physiological roles of Mn, MnO_2_ NPs are expected to play promising roles in the development of novel nanomedicines.

Taking the advantages of the advancing biological activities of Mn, MnO_2_ NPs also exert promising biological and immunological activity, and can also be used as a drug carrier for targeted drug delivery. The MnO_2_ nano preparations can enhance the drug permeability and retention effect, and also enhance the stability of the drugs in the complicated physiological environment and allow the accumulation of drugs at the targeted site, which always lead to enhanced efficacy by combining the drug effects and biological activity of MnO_2_ NPs (131).

More importantly, MnO_2_ NPs also possess strong immunological regulation effects to regulate both innate and adaptive immunity. In tumor, hypoxia not only limits the efficacy of RT, PT, etc., but also causes the accumulation of immunosuppressors in tumor and limits the infiltration of immune cells. MnO_2_ NPs exert the activity of peroxidase to produce O_2_ *in situ* in the tumor to improve the immunosuppression while reshaping tumor microenvironment, and further enhance the anti-tumor immunity by promoting the infiltration of innate immune cells (7). MnO_2_ can relieve hypoxia at the tumor site by REDOX with excess H_2_O_2_ and GSH in TME, and enhance the antitumor efficacy of RT (44), PT (17, 41), SDT (45), CDT (23, 32) and their synergistic treatment (5, 17). After the entry into cells, MnO_2_ NPs can interact with endogenous H_2_O_2_ to increase OH· levels, by depleting GSH and mediating Fenton-like reactions, thereby enhancing the antitumor efficacy of chemodynamic therapy and chemotherapy (32).

During the interactions between MnO_2_ NPs and GSH or H_2_O_2_, Mn^2+^ can be released to show multiple biological functions. The obtained Mn^2+^ can directly bind with cGAS to enhance their enzyme activity and their sensitivity to dsDNA. The binding of Mn^2+^ with cGAS could also activate STING by enhancing the affinity between cGAMP and STING to promote the CGAS-STING signaling pathway, which would induce the production of IFN-I to promote innate immunity. IFN-I can further promote the mature activation of infiltrating APCs to trigger adaptive immunity, which therefore introduces the potential of Mn^2+^ to be used as an immune adjuvant (9, 38, 107, 128).

Mn^2+^ released by MnO_2_ NPs after catalyzing H_2_O_2_ can mediate Fenton-like reaction to produce OH·, which induces oxidative stress in tumor cells and acute inflammatory response, thus enhancing DC maturation and TNF-α production to further promote innate immune responses (32, 55). MnO_2_ NPs can also indirectly improve adaptive immunity by producing *in situ* O_2_ to overcome immune escape caused by HIF-1α-dependent PD-L1 expression in tumor cells under hypoxia conditions (121). MnO_2_ NPs can also be used as a unique nutrition-responsive ICD inducer, selectively inducing ICD in nutrition-deficient tumor cells to regulate adaptive immunity (110).

Based on these properties, MnO_2_ NPs have shown great potentials as novel immune adjuvants and agonists due to its various biological activities and immune regulatory functions. Due to the development of Mn^2+^ -based adjuvants, the use of MnO_2_ NPs for potential adjuvant uses might also attract increasing attentions. Additionally, more and more researchers are working on how to integrate MnO_2_ NPs into multiple mode agents for diagnostic and therapeutic uses simultaneously. The development of such multiple mode agents based on MnO_2_ NPs might introduce more important advances, including its immunotherapy potentials, multi-mode synergistic therapy, and MRI guided chemotherapy or immunotherapy.

However, in order to apply MnO_2_ NPs for effective clinical uses, its biocompatibility and toxicity, and their associated metabolism and degradation are the most important issues that need to be further investigated. And among these issues, biocompatibility and toxicity are the most urgent issues for the further uses of MnO_2_ NPs. Some studies have proposed (41, 132) that the wrap of MnO_2_ NPs with HSA could introduce MnO_2_ NPs better biocompatibility and appropriate size, which is conducive to enhancing the permeability and retention effect of nanoparticles. In addition, studies (18, 38) have shown that loading MnO_2_ NPs with GC or PEG can also enhance their biocompatibility and improve their colloid stability More studies have pointed out that polydopamine (PDA) is a “connector” of functional materials due to its strong adhesion and chemical reactivity, and MnO_2_ NPs can also be connected to PDA with good biocompatibility (133). The encapsulation of MnO_2_ NPs in a carrier constructed with HA could also make MnO_2_ NPs more biocompatible for cancer treatment (63). Although lots of attempts have been made to improve the biocompatibility and reduce the toxicity of MnO_2_ NPs, more attentions are still needed to promote the final clinic uses of MnO_2_ NPs. Moreover, the systemic metabolism and degradation behaviors and mechanisms are also need to be further evaluated. On the premise of maintaining the therapeutic effect of biological system, attention should be paid to reducing or reducing the toxicity *in vivo* and speeding up its biodegradability.In addition to the primary issue of biocompatibility, the absence of a perfect preparation method is also the key hindering the clinical application of MnO_2_ NPs.Currently, there are many methods to prepare nanomaterials, which have their own advantages but also their own obvious disadvantages, such as difficult preparation methods and high cost, difficult to control the consistency of nanomaterials, potential dangers and environmental pollution (134, 135). Therefore, it remains a major challenge to explore a simple, low-cost, safe and environmentally friendly method for the controlled synthesis of MnO_2_ NPs.

Thus, although MnO_2_ NPs have shown promising application in plenty of biological and medical uses, such as their excellent immunoregulation effects, there are still many obstacles that affect their clinical use, which requires more researches and investigations. However, we believe that as one of the most functional nanomaterials with strong immunoregulation activities, MnO_2_ NPs would play more and more important roles for the development of novel vaccines or immunotherapies, which would finally benefit the more effective disease precaution and treatments.

## Author contributions

YuH and YR drafted this manuscript, YM, DC, TZ, SF, WL, YiH, HL helped to revise the manuscript, J-FX, JP and BZ helped to revise the manuscript and were responsible for leading this work. All authors contributed to the article and approved the submitted version. 
